# Enhancing spinal cord injury repair through PTCH1-mediated neural progenitor cell differentiation induced by ion elemental-optimized layered double hydroxides

**DOI:** 10.1016/j.mtbio.2025.101918

**Published:** 2025-05-29

**Authors:** Feng Zhang, Xinghao Pan, Kaikai Zhang, Shuhan Liu, Danni Yu, Jingjing Su, Tong Zhu, Song Chen

**Affiliations:** a Department of Orthopedics, Centre for Leading Medicine and Advanced Technologies of IHM, The First Affiliated Hospital of USTC, Division of Life Sciences and Medicine, University of Science and Technology of China, Hefei, Anhui, 230001, China; b Department of Orthopedics, Quzhou People's Hospital, The Quzhou Affiliated Hospital of Wenzhou Medical University, No.100, Minjiang Avenue, Quzhou, 324000, Zhejiang, China; c Department of Laboratory Medicine, Shanghai Pulmonary Hospital, Tongji University School of Medicine, Shanghai, China; d The First Affiliated Hospital of Anhui Medical University, Hefei, 230001, China; e People's Hospital of Qianxinan Prefecture, Guizhou province, 562400, China; f Department of Medicine, Lady Davis Institute-Jewish General Hospital, McGill University, Montreal, QC, Canada

**Keywords:** Layered double hydroxide, Neural progenitor cells, Embryonic stem cells, Cell membrane receptor, Spinal cord injury

## Abstract

Spinal cord injury (SCI) is associated with profound neurological impairments, and to date, efficacious therapeutic interventions remain elusive. Embryonic stem cells (ESCs) possess the totipotent capacity to differentiate into specific neuronal cell types under the influence of appropriate extrinsic signals. Notably, their induction into neural progenitor cells (NPCs) holds particular promise. These NPCs are capable of self-renewal and can differentiate into all neuronal cell types, exhibiting the ability to migrate and integrate into damaged areas of the central nervous system (CNS), thereby emerging as an ideal therapeutic strategy for neurological disorders. Layered double hydroxides (LDHs), with their lamellar architecture, are biocompatible and possess anion-exchange attributes, making them prominent in drug and nucleotide delivery for tissue engineering. Nevertheless, the investigation into the intrinsic biological effects of LDHs are rarely reported. Our research demonstrates that MgFe-LDH and MgAl-LDH promote NPCs differentiation in a dose-dependent manner, and MgAl-LDH is superior to MgFe-LDH in promoting NPCs differentiation. RNAseq revealed that the promoted NPCs differentiation by nanoparticles was primarily associated with the interaction between nanoparticles and transmembrane protein PTCH1. Furthermore, we performed PTCH1 knockdown in NPCs and observed a significant impact on the MgAl-LDH-induced NPCs differentiation. In *vivo*, MgAl-LDH-pretreated NPCs implantation significantly enhances the behavioral and electrophysiological performance of SCI mice, and neurons clearly observed in the lesion sites of MgAl-LDH-pretreated NPCs group. This work provides novel strategies and a theoretical foundation for the research on nanomaterial regulation of stem cells fate and neural regenerative repair.

## Graphical abstract

Fig. 9. Schematic diagram of MgAl-LDH-pretreated NPCs in repairing spinal cord injury

**Figure:**
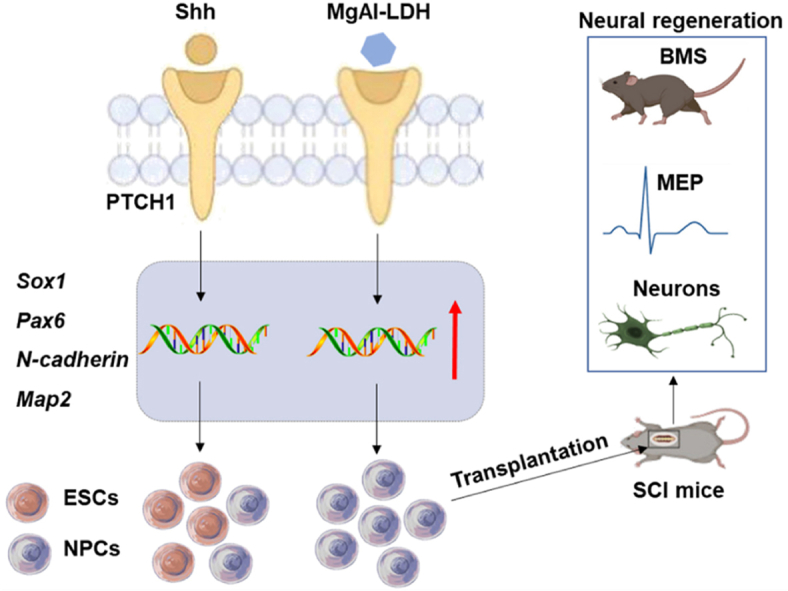

## 1 Introduction

Spinal cord injury (SCI) represents a profound neurological impairment characterized by the impairment of sensory and motor faculties [1,2]. Post-SCI, a deleterious inflammatory milieu emerges, which, coupled with the cascade of primary and secondary neuronal demise as well as the dearth of neurotrophic factors, exacerbates the intrinsic regenerative capacity of the spinal cord [3]. Current clinical interventions, including pharmacological neurotrophic agents, surgical interventions, and rehabilitative exercises, have yielded suboptimal outcomes [4]. Consequently, the therapeutic management of SCI continues to pose a formidable challenge.

Embryonic stem cells (ESCs) are pluripotent entities capable of differentiating into a variety of neural cell types under specific culture conditions, including neural progenitor cells (NPCs), dopaminergic neurons, motor neurons, and oligodendrocyte precursor cells [[5], [6], [7]]. NPCs possess the potential to differentiate into all neural cell lineages and can be transplanted into damaged regions of the central nervous system for regenerative purposes [8,9]. Consequently, elucidating the underlying mechanisms that govern the differentiation trajectory of ESCs into NPCs is imperative for advancing our understanding of neural development and therapeutic applications.

Nanomaterials have emerged as innovative neuroregulatory tools capable of precisely modulating cell fate and neuronal activity [[10], [11], [12], [13]]. Recent investigations have demonstrated that functional biomaterials possess significant potential in addressing the challenges associated with neural fate regulation [14,15]. Nonetheless, the ability to direct specific differentiation pathways remains constrained, and the underlying molecular mechanisms governing these cell-material interactions are not yet fully elucidated. Stem cell surface receptors, which are adept at sensing extracellular cues, play a pivotal role in the regulation of stem cell fate [16]. By modulating these cell membrane receptors, it is possible to influence axonal guidance, synaptic formation, and neuronal regeneration [[17], [18], [19]], thereby offering novel therapeutic avenues for neurodegenerative diseases.

The choice of appropriate nanomaterials, along with their morphology and properties, should be guided by the specific application requirements and the characteristics of the target cells. The physicochemical properties of nanomaterials play a crucial role in determining their efficacy in cell fate regulation [20,21]. Layered double hydroxide (LDH) nanoparticles, a type of anionic hydrotalcite-like clay, possess excellent biocompatibility and stable physicochemical properties, allowing their interaction with cell membranes to exert biological effects [[22], [23], [24]]. In recent years, LDH has gained attention in neural regeneration field, and previous studies have explored its potential in drug delivery, tissue engineering, and spinal cord injury repair [25]. For instance, Pang et al. demonstrated LDH's effective drug loading and sustained release capacity in neural tissue engineering [26]. Zhang et al. reported the application of LDH-based scaffolds in promoting spinal cord injury repair by supporting cell adhesion and growth [27]. Moreover, LDH nanoparticles had been demonstrated to effectively improve the immune microenvironment by activating transforming growth factor-β receptor [28]. However, the application of LDH in directly regulating cell membrane receptors to influence stem cell differentiation, particularly in the context of ESCs to NPCs, is still in their infancy. Further research is essential to comprehensively understand the interactions between LDH nanomaterials and cell membrane receptors, and to assess their potential in biomedical applications.

The main aim of this work was to explore how different metal-element LDHs influence the differentiation of ESCs into NPCs by interfering with cell membrane receptors, and to evaluate the subsequent effects on spinal cord injury repair. In this study, we thoroughly investigated the roles of two different metal-element LDH (MgFe-LDH and MgAl-LDH) in the differentiation of ESCs into NPCs. The incorporation of different metal ions in LDH can alter its physicochemical properties, thereby influencing cell fate to varying degrees [[29], [30], [31]]. Our results demonstrated that MgAl-LDH nanoparticles significantly upregulated the level of cell membrane receptor PTCH1 compared to MgFe-LDH, thereby more effectively promoting ESCs-derived NPCs differentiation. Furthermore, MgAl-LDH-pretreated NPCs implantation significantly enhanced the behavioral and electrophysiological performance in SCI mice, and promoted neural regeneration in the lesion sites. An in-depth exploration of the interactions between cell membrane receptors and LDH nanoparticles will contribute to the development and optimization of novel materials, enhancing our understanding of the application of nanomaterials in stem cell fate regulation and introducing new strategies for SCI recovery.

## 2 Methods & materials

### 2.1 Reagents and materials

Mg(NO_3_)_2_·6H_2_O, Fe(NO_3_)_3_·9H_2_O, Al(NO_3_)_3_·9H_2_O and NaOH were bought from Sinopharm Group Co. Ltd. (Shanghai, China). Dulbecco's modified eagle medium (DMEM), Glasgow minimum essential medium (G-MEM), Neurobasal, DMEM/F12 fetal bovine serum (FBS), B27, N2, NT3, BDNF, nonessential amino acids (NEAA), Glutamax, penicillin and streptomycin, sodium pyruvate, fluorescein isothiocyanate (FITC) were purchased from Thermo Fisher Scientific (MA, USA). *β*-mercaptoethanol, paraformaldehyde (PFA), Triton X-100, and goat serum were obtained from Sigma-Aldrich(MO, USA). LIF was purchased from Millipore (MA, USA).

### 2.2 Synthetization and characterization of LDH

MgFe-LDH and MgAl-LDH nanoparticles were fabricated through a coprecipitation method followed by hydrothermal treatment [22]. The procedure involved dissolving 1.538 g of Mg(NO_3_)_2_·6H_2_O and 0.606 g of Fe(NO_3_)_3_·9H_2_O in 20 mL of deionized water. This solution was then introduced into a vigorously stirred NaOH solution maintained at 60 °C for a 30 min reaction. The resulting precipitate was collected and subjected to hydrothermal conditions at 100 °C for 16 h. Following centrifugation and thorough washing with deionized water, MgFe-LDH nanoparticles were successfully obtained. For the synthesis of MgAl-LDH nanoparticles, 0.75 g of Al(NO_3_)_3_·9H_2_O was used as a substitute for 0.606 g of Fe(NO_3_)_3_·9H_2_O, while the rest of the procedure remained unchanged.

The morphology of the nanoparticles was characterized using transmission electron microscopy (TEM, Talos F200X, FEI, Thermo Fisher) and scanning electron microscopy (SEM, Gemini 300, Zeiss, Germany). Fourier transform infrared (FTIR) spectroscopy was employed to obtain the spectra of the nanoparticles using a Bruker FTIR spectrometer (Vertex 70, Bruker, Germany). The crystalline phases of MgFe-LDH and MgAl-LDH were identified through X-ray diffraction (XRD, Ultima IV, Rigaku, Japan). The surface zeta potential of the nanoparticles was measured with a Nano Zetasizer (Nano ZS90, Malvern Panalytical, UK) ICP-OES (ICP 2100, PerkinElmer, Massachusetts) was used to detect the metal ion release of MgAl–LDH and MgFe–LDH.

### 2.3 Cell culture and viability assay

mESCs were procured from the National Collections of Authenticated Cell Cultures and maintained on feeders in mESC medium, which comprised DMEM supplemented with 15 % FBS [18,29,32], 1 % penicillin-streptomycin, 1 % NEAA, 1 % GlutaMAX, 1 % sodium pyruvate, 0.1 mM β-mercaptoethanol, and 1000 U mL^−1^ LIF. The mESCs were subcultured every 2–3 days, with daily medium replacement. For NPCs differentiation, mESCs were seeded at a density of 5 × 10^4^ cells mL^−1^ in ultralow attachment plates containing NPCs medium (G-MEM supplemented with 8 % knockout serum replacement, 1 % penicillin-streptomycin, 1 % NEAA, 1 % GlutaMAX, 1 % sodium pyruvate, and 0.1 mM β-mercaptoethanol). The spheres were passaged and counted every 3 days, with concurrent medium replacement. NPCs were exposed to nanoparticles on the 3rd day of NPCs differentiation (when spheres formed)

For neuronal differentiation, NPCs were plated into 24-well plates, and cultured in neuronal medium (50 % neurobasal, 50 % DMEM/F12, 1 % penicilin and streptomycin, 2 % B27, 1 % N2, 10 ng mL^−1^ NT3 and 10 ng mL^−1^ BDNF). The medium was refreshed every 2 days. Following a 21 days differentiation period, the cells exhibited typical neuronal morphology, and the neurons were then detected by qPCR and immunofluorescence.

The CCK-8 assay (APExBIO Technology, Houston) was employed to evaluate the cell viability of NPCs treated with nanoparticles, following the manufacturer's protocol. Briefly, post-trypsinization, the cells were harvested, and NPCs were seeded into gelatin-coated 96-well plates at a density of 5 × 10^3^ cells per well and incubated overnight. Wells containing only medium served as the blank control group. Subsequently, the medium was replaced with NPCs medium containing nanoparticles at concentrations of 2.5, 5, 10, 20 μg mL^−1^. After 72 h, NPCs were incubated with 10 μL of CCK-8 solution for an additional 2 h. Absorbance was measured at 450 nm using a microplate reader (Multiskan FC, Thermo Fisher), and cell viability for each group was calculated relative to the control group.

### 2.4 Lactate dehydrogenase release assay

A lactate dehydrogenase assay kit (KeyGEN BioTECH, Nanjing) was utilized to assess cell membrane integrity. Cells were seeded overnight and subsequently treated with nanoparticles for 72 h. Following treatment, 100 μL of the supernatant was transferred to a new 96-well plate containing 100 μL of working solution and incubated at room temperature for 30 min. Absorbance was measured at 490 nm using a microplate reader.

### 2.5 Cell cycle analysis and cellular uptake analysis

Cell cycle analysis was conducted using a FACScan flow cytometer, following the manufacturer's protocol. NPCs were subjected to 48 h exposure with gradient concentrations (0–20 μg mL^−1^) of MgFe-LDH and MgAl-LDH. Following treatment, cellular suspensions were prepared through enzymatic dissociation and subsequently processed through sequential staining protocols: initial fixation in 500 μL of ice-cold 70 % ethanol at 4 °C for 4 h, followed by centrifugation-assisted ethanol removal (2000 rpm, 3 min). The samples were then resuspended in 450 μL PI solution, and co-incubated with 50 μL RNase An under light-protected conditions for 30 min. The cells were then filtered through a 200-mesh sieve, and 1.0 × 10^4^ cells of each group were analyzed.

To detect the uptake efficiency of NPCs after LDH treatment, LDH was incubated 1:1 with FITC overnight. Subsequently, NPCs were cultured with LDH-FITC for 24 h. For cellular uptake analysis, LDH-treated NPCs were collected, and then transferred to a flow tube through a 300-mesh filter. Blank cells were the control group. The fluorescence intensity of FITC was determined by flow cytometry.

### 2.6 Cytoskeleton staining assay

FITC was conjugated with 20 μg mL^−1^ LDH in PBS under dark conditions at 4 °C overnight to ensure optimal binding. LDH-treated spheres were fixed with 4 % PFA, and then the cytoskeleton was labeled using ActinRed (KeyGEN BioTECH, Nanjing), and cell nuclei were stained with DAPI (Sigma-Aldrich).

### 2.7 Transcriptomic analysis

The transcriptomic profiles of NPCs subjected to MgFe-LDH and MgAl-LDH treatments were scrutinized via RNA-seq to elucidate the impact of these nanoparticles on NPCs differentiation. Differentially expressed genes (DEGs) between the nanoparticles-treated groups and the control group were identified. Subsequent analyses, including heatmap visualization, Kyoto Encyclopedia of Genes and Genomes (KEGG) pathway analysis, Gene Ontology (GO) enrichment analysis, Gene Set Enrichment Analysis (GSEA), and protein–protein interaction (PPI) network construction, were conducted utilizing R software.

### 2.8 Immunofluorescent staining

Spheres were fixed with 4 % PFA, permeabilized with 0.5 % Triton X-100, and blocked with 7.5 % goat serum. Then samples were incubated with the primary antibody at 4 °C overnight. Following incubation with fluorescent secondary antibodies and DAPI staining, samples were observed using a Leica confocal microscope. The primary antibodies used were as follows: SOX1, PAX6, N-CADHERIN, MAP2, GFAP and PTCH1 were purchased from Abcam (CA, USA), and fluorescent secondary antibodies were purchased from Millipore (MA, USA).

For the immunofluorescence analysis of tissue specimens, the protocol was executed as delineated: Mice were subjected to isoflurane-induced anesthesia, followed by intracardiac perfusion with PBS and subsequent fixation with a 4 % paraformaldehyde solution. The spinal cord segment (∼1.5 cm) encompassing the lesion site was meticulously dissected, further fixed in a 4 % paraformaldehyde solution, and subjected to dehydration using a 30 % sucrose solution. Coronal sections, 10 μm in thickness, were meticulously sectioned using a cryostat. The sections were incubated with 0.25 % Triton X-100 for 10 min to facilitate samples permeabilization. Subsequently, the sections were incubated with primary antibodies at 4 °C overnight after blocking. Following incubation with fluorescent secondary antibodies and staining with DAPI, the specimens were examined and imaged using confocal microscopy.

### 2.9 Colocalization of PTCH1 and nanoparticles

NPCs were incubated with the FITC-labeled LDH (LDH-FITC) for 24 h. Subsequent immunofluorescence assays were conducted to observe the colocalization of PTCH1 and nanoparticles.

### 2.10 Genes knockdown in NPCs

The plasmid designed for *Ptch1* knockdown was purchased from Generay Biotechnology. Lipofectamine 3000 was employed to transfect the plasmid into NPCs. Post-transfection, the NPCs were subjected to puromycin selection for 48 h to ensure successful integration. qPCR and immunofluorescence analysis were subsequently employed to evaluate the knockdown efficiency of *Ptch1*.

### 2.11 Animals

All animal studies were conducted with the approval of the Animal Welfare Committees at Shanghai Pulmonary Hospital and strictly adhered to the ethical guidelines established by these committees. Female C57BL/6 mice, weighing approximately 20 g and at eight weeks of age, were procured from Shanghai JieSiJie Laboratory Animal Company. Prior to the commencement of the experiments, the mice were permitted to acclimate to the environmental conditions of their housing for a minimum period of one week to ensure their well-being and reduce stress-induced variability in experimental outcomes.

### 2.12 Surgical procedure and experimental design

A clamp SCI model was used in this work. The surgical procedure was performed according to a previous report [33]. Following T9 vertebral laminectomy, the dorsal spinal cord was surgically exposed while preserving dural membrane integrity. A precision-engineered microsurgical instrument was stereotactically mounted on a calibrated positioning system. The device's distal tips were bilaterally aligned with the T9 spinal cord midline under microscopic guidance. Controlled vertical displacement was applied until definitive osseous contact was established with the ventral vertebral body (total displacement: 0.5 mm). Complete forceps occlusion was achieved with zero-gap interface between instrument tips and neural tissue. Mechanical compression was maintained for 3 s under continuous physiological monitoring. Polypropylene sutures were used for post-procedural closure. The mice were systematically randomized and divided into three distinct groups for comparative analysis: (1) SCI group, comprising mice with SCI that received no additional interventions; (2) NPCs group, consisting of SCI mice into which NPCs (1 × 10^5^/2 μL) were orthotopically engrafted at the central site of lesion; (3) LDH-pretreated NPCs group, encompassing SCI mice that underwent implantation of NPCs (1 × 10^5^/2 μL) subjected to LDH pretreatment. The specific approval number is K24-202Y.

### 2.13 Behavioral assessment

The open-field locomotor performance was rigorously assessed using the BMS, with evaluations conducted weekly over an extended period of eight weeks, in strict accordance with the double-blind methodology. The locomotor capabilities of the bilateral hindlimbs were ascertained exclusively through the BMS scoring system and subsequently averaged to derive a composite measure of motor function.

### 2.14 Electrophysiological studies

Electrophysiological assessments were conducted eight weeks postoperatively, adhering to the methodologies delineated in a preceding study [34]. MEPs serve as a comprehensive metric for the evaluation of an animal's locomotor capabilities and are indicative of the extent of neurological restoration following spinal cord injury.

### 2.15 mRNA extraction and RT-qPCR analysis

Total RNA was extracted from the samples using the SPARKeasy Cellular RNA Extraction Kit (Sparkjade Biotechnology, Shandong). Reverse transcription was subsequently performed using the SPARKscript II RT Plus kit (with gDNA Eraser) (Sparkjade Biotechnology, Shandong). cDNA was subjected to qPCR analysis on a LightCycler LC96 real-time PCR system (Roche) utilizing the 2 × SYBR Green qPCR Mix (Sparkjade Biotechnology, Shandong). The primer sequences used for this work were listed in Table S1.

### 2.16 Haematoxylin and eosin (H&E) staining

Histological examination of visceral tissues was conducted utilizing the H&E staining. The procedure commenced with the infiltration of tissues in paraffin, facilitating subsequent processing. The embedded samples were sectioned into 10 μm-thick slices using a precision microtome. The sections were then subjected to a standardized staining protocol to elucidate tissue morphology. Subsequent to staining, images were captured using a microscope.

### 2.17 Statistical analysis

All data are expressed as the mean ± standard deviation (SD). Statistical significance was assessed using one-way or two-way analysis of variance (ANOVA). All statistical analyses were performed using GraphPad Prism 9 software. The symbols ∗, ∗∗, and ∗∗∗ denote p-values of less than 0.05, 0.01, and 0.001, respectively. The symbols “ns” means there was no significant difference between the two groups.

## 3 Results & discussion

### 3.1 Characterization of MgFe-LDH and MgAl-LDH

**Fig. 1:**
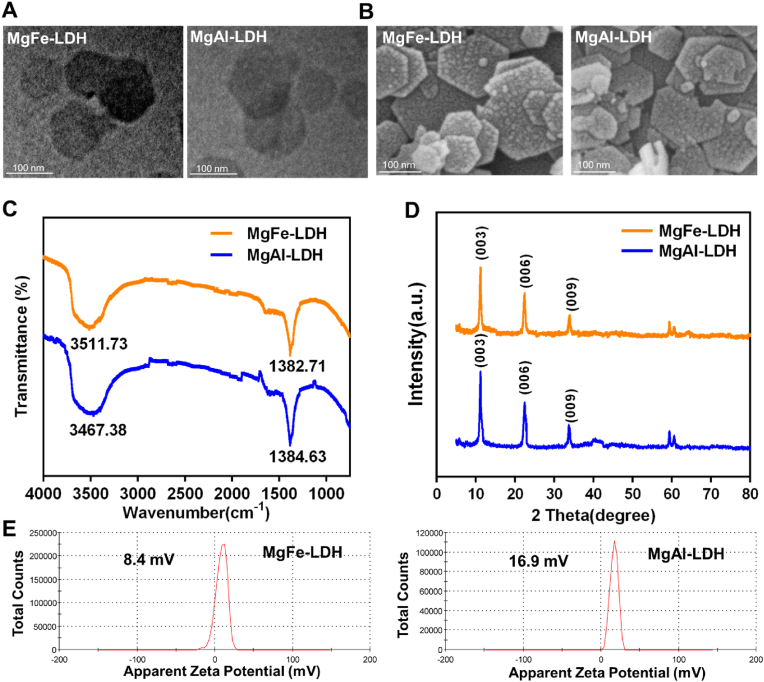
Characterization of MgFe-LDH and MgAl-LDH. A) TEM images of MgFe-LDH and MgAl-LDH; B) SEM images of MgFe-LDH and MgAl-LDH; C) FTIR spectra of MgFe-LDH and MgAl-LDH; D) XRD patterns of MgFe-LDH and MgAl-LDH; E) Mean Zeta potential of MgFe-LDH and MgAl-LDH.

The morphological characteristics of the obtained MgFe-LDH and MgAl-LDH were meticulously analyzed using TEM and SEM. As depicted in the TEM images (Fig. 1A), both MgFe-LDH and MgAl-LDH exhibited a distinct layered hexagonal morphology. Complementary SEM images (Fig. 1B) further corroborated this observation, revealing a consistent hexagonal layered structure on the morphology of both MgFe-LDH and MgAl-LDH [23]. The FTIR spectra (Fig. 1C) of MgFe-LDH and MgAl-LDH displayed prominent stretching vibration peaks of O-H at 3511.73 cm^−1^ and 3467.38 cm^−1^, respectively. Additionally, the stretching vibration peaks of NO_3_^−^ were observed at 1382.71 cm^−1^ and 1384.63 cm^−1^, respectively. XRD analysis (Fig. 1D) identified characteristic diffraction peaks at (003), (006), and (009) for both MgFe-LDH and MgAl-LDH, confirming the successful construction of the layered structures [35]. Zeta potential analysis (Fig. 1E) revealed that MgFe-LDH and MgAl-LDH possessed an average surface charge of +8.4 mV and +16.9 mV, respectively. The positive surface charge is conducive to electrostatic interactions with anionic cell membranes, facilitating potential biomedical applications [36].

### 3.2 Biocompatibility evaluation of MgFe-LDH and MgAl-LDH in NPCs

**Fig. 2:**
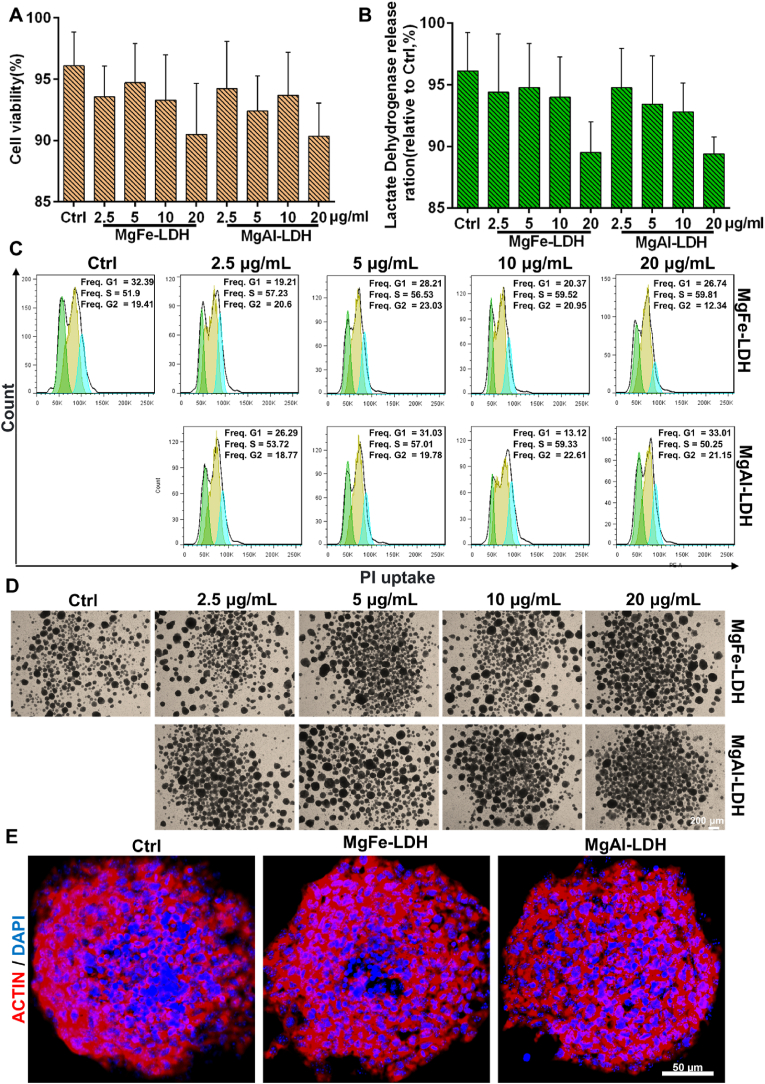
Nanoparticles biocompatibility with NPCs. A) Cell survival analysis of NPCs treated with nanoparticles at different doses for 72 h; B) Detection of lactic dehydrogenase release from NPCs treated with nanoparticles at different doses for 72 h; C) FACS detection of the cell cycle variation of NPCs treated with nanoparticles at different doses for 72 h; D) Cell morphology of NPCs treated with nanoparticles at different doses for 72 h; E) Cytoskeleton of 20 μg mL^−1^ nanoparticles-treated NPCs.

The viability of NPCs treated with MgFe-LDH and MgAl-LDH for 72 h was evaluated using a Cell Counting Kit-8 (CCK-8) assay. The results indicated no significant differences in cell viability between the control and LDH-treated groups at different concentrations (Fig. 2A). Considering that LDH can interact with the negatively charged cell membrane [37], the release of lactate dehydrogenase from the cytoplasm is measured as an indicator of cell membrane integrity post-nanoparticles treatment (Fig. 2B). In comparison to the control group, the LDH-treated groups at various concentrations exhibited no significant alterations in lactic dehydrogenase release, indicating that these nanoparticles had no impact on cell membrane integrity. As illustrated in Fig. 2D, flow cytometry analysis revealed that treatment with MgFe-LDH and MgAl-LDH for 72 h did not significantly induce G2/M cell cycle arrest. Furthermore, the spherical morphology of NPCs remained largely unchanged after 72 h of incubation with varying concentrations of MgFe-LDH and MgAl-LDH (Fig. 2D), and both MgFe-LDH and MgAl-LDH had no effect on the morphology of cytoskeleton (Fig. 2E). The above results validated the biocompatibility of MgFe-LDH and MgAl-LDH nanoparticles, affirming their appropriateness for NPCs culture. Furthermore, Fig. S2A showed that a more positive FITC signal was observed in the MgAl LDH than in the MgFe LDH group, which may imply that MgAl LDH was more easily taken up by cells and had a higher accumulation in cells. As shown in Fig. S2B, the release of divalent and trivalent metal ions was stable after 24 h, and the maximum Mg^2+^ release of MgAl-LDH and MgFe-LDH was similar at 22.3 % and 22.6 %, while the maximum Al^3+^ and Fe^3+^ release showed significant difference at 31.2 % and 17.5 %, respectively, consistent with previous reports [30].

### 3.3 MgAl-LDH is superior to MgFe-LDH in supporting NPCs differentiation

**Fig. 3:**
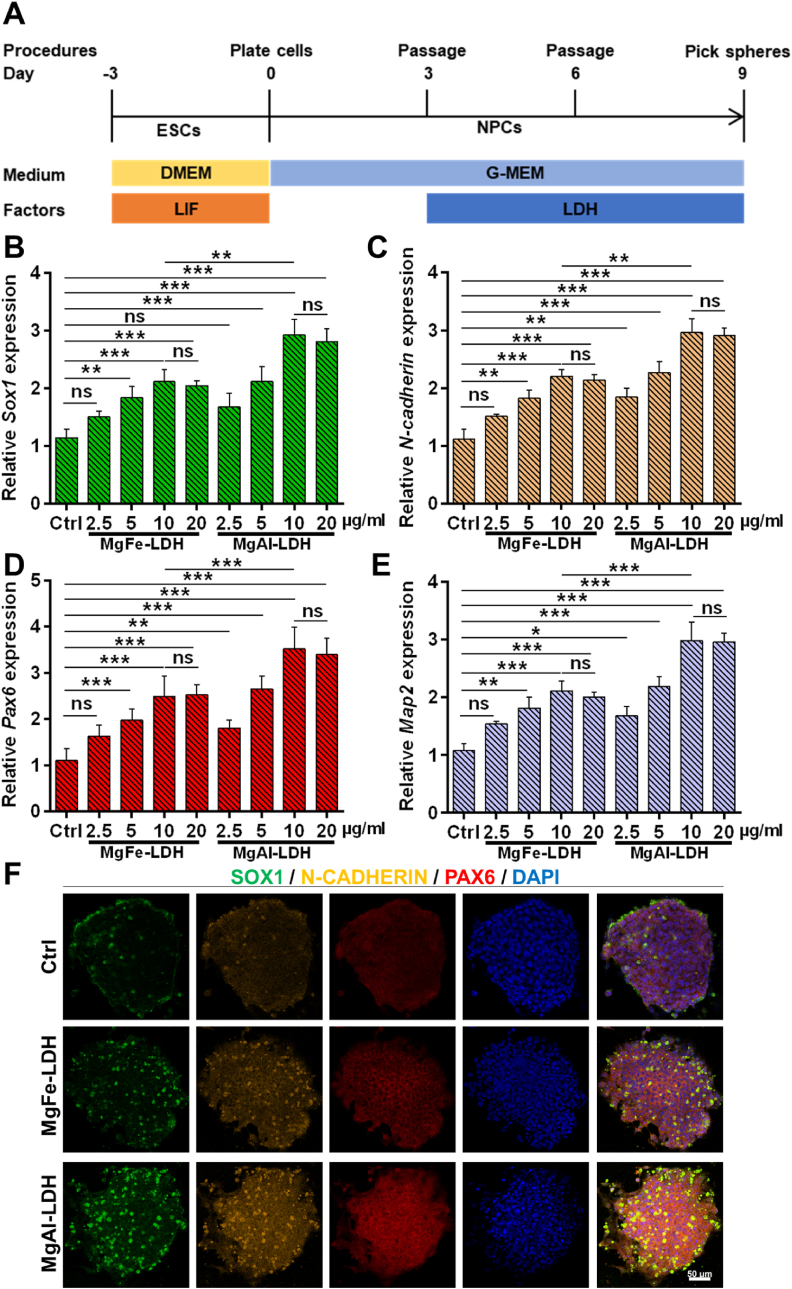
The improved NPCs differentiation by LDH. A) Overview of the NPCs differentiation strategy; B-E) mRNA level of *Sox1*, *N-cadherin*, *Pax6*, and *Map2* in NPCs treated with nanoparticles at different doses detected by qPCR; F) Immunofluorescence analysis of the protein level of SOX1, N-CADHERIN, and PAX6 in NPCs treated with nanoparticles. The data are presented as the mean ± SD (n = 3, ∗∗∗p < 0.001, ∗∗p < 0.01, ∗p < 0.05, ns means there was no significant difference between the two groups).

To elucidate the role of MgFe-LDH and MgAl-LDH in the NPCs differentiation, nanoparticles were added into the NPCs differentiation system on the 3rd day of the differentiation period, and the spheres treated with MgFe-LDH and MgAl-LDH were subsequently harvested on the 9th day for qPCR and immunofluorescence analysis (Fig. 3A). As illustrated in Fig. 3B–E, the mRNA levels of NPC markers (*Sox1*, *Pax6*, *N-cadherin*, and *Map2*) in the LDH-treated groups exhibited significant upregulation compared to the control group, with gene expression demonstrating a concentration-dependent response, and differentiation efficiency plateaued at 20 μg mL^−1^. Notably, the MgAl-LDH group displayed elevated mRNA levels of *Sox1*, *Pax6*, *N-cadherin*, and *Map2* compared to the MgFe-LDH group at equivalent dosages, suggesting a superior efficacy of MgAl-LDH in promoting NPCs differentiation. The concentration of 10 μg mL^−1^ is commonly utilized for LDH in regulating stem cell fate [29,32,38], and this concentration was subsequently adopted in further studies. Concurrently, protein expression was assessed (Fig. 3F), and immunofluorescence analysis revealed that the fluorescence intensity of SOX1, PAX6, and N-CADHERIN in both MgAl-LDH and MgFe-LDH treatments exhibited a pronounced elevation (Fig. S1). The MgAl-LDH group demonstrated stronger fluorescence intensity of SOX1, PAX6, and N-CADHERIN than the MgFe-LDH group. Collectively, these findings uncovered the enhanced capability of MgAl-LDH nanoparticles in facilitating NPCs differentiation compared to MgFe-LDH nanoparticles.

### 3.4 Transcriptomic analyses

**Fig. 4:**
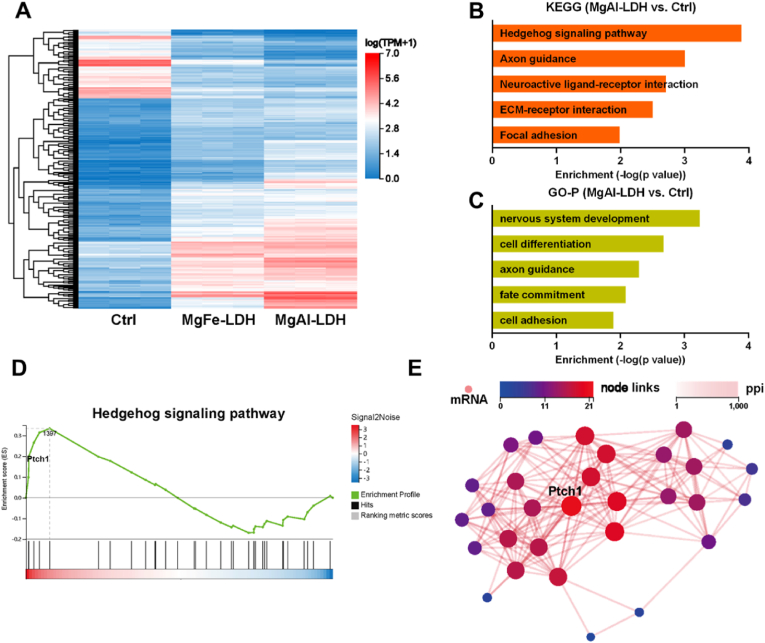
RNA-seq revealed the promoted NPCs differentiation by nanoparticles. A) Heatmap of DEGs; B) KEGG pathway enrichment analyses; C) GO enrichment analyses; D) GSEA between the MgAl-LDH group and control group; E) PPI analysis of MgAl-LDH regulated significant genes.

Our study had demonstrated that MgAl-LDH nanoparticles could facilitate the differentiation of NPCs, while the underlying mechanisms remained to be elucidated. RNA-seq were conducted to identify critical genes and pathways by analyzing differentially expressed genes (DEGs). The heatmap (Fig. 4A) illustrated a significant distinction in expression profiles between the control and MgAl-LDH groups. Subsequently, the 280 most significant DEGs (p < 0.05 and log_2_ fold change>2) in heatmap from Fig. 4A were selected for further analysis. KEGG pathway analysis revealed that these DEGs were predominantly enriched in the hedgehog signaling pathway, axon guidance, neuroactive ligand-receptor interaction, ECM-receptor interaction, and focal adhesion (Fig. 4B). DEGs between the MgFe-LDH and MgAl-LDH groups were also predominantly enriched in the hedgehog signaling pathway (Fig. S3A). GO terms were associated with nervous system development, cell differentiation, axon guidance, fate commitment, and cell adhesion (Fig. 4C). DEGs between the MgFe-LDH and MgAl-LDH groups were also predominantly associated with cell differentiation, nervous system development, and fate commitment (Fig. S3B). Furthermore, the membrane receptor PTCH1 exhibited significant alterations in the GSEA of the hedgehog signaling pathway between the MgAl-LDH and control/MgFe-LDH groups (Fig. 4D and S3C). As a component of the hedgehog signaling pathway, PTCH1 is essential for regulating cell differentiation [39]. PTCH1 is a crucial signaling protein that plays a key role in neural development [40]. During neural development, PTCH1 influences the generation of neurons and glial cells by modulating the behavior of neural stem cells and progenitor cells [39]. Studies have shown that mutations or dysfunctions in PTCH1 can lead to various neurodevelopmental disorders [41,42]. The PPI analysis of MgAl-LDH and the other two groups indicated that PTCH1 played a pivotal role in promoting NPCs differentiation regulated by MgAl-LDH nanoparticles (Fig. 4E and S3D).

### 3.5 LDH promotes NPCs differentiation by activating hedgehog signaling pathways through binding with transmembrane protein PTCH1

Nanomaterials can interact with specific receptors on the cell membrane, thereby activating or inhibiting signal transduction pathways. For instance, gold nanoparticles (AuNPs) bind to epidermal growth factor receptors (EGFR), activating the downstream MAPK/ERK signaling pathway, which promotes cell proliferation and differentiation [17]. Silver nanoparticles (AgNPs) interact with integrin receptors, affecting cell adhesion and migration, thus regulating stem cell differentiation [43]. Iron oxide nanoparticles (IONPs) bind to transferrin receptors (TfR), facilitating nanoparticle internalization and intracellular signal transduction, thereby influencing cell proliferation and differentiation [44]. Silicon dioxide nanoparticles (SiNPs) interact with Toll-like receptors (TLR), triggering immune and inflammatory responses, which in turn affect stem cell differentiation [45]. Carbon nanotubes (CNTs) interact with integrin receptors, modulating cytoskeletal reorganization and signal transduction, thereby impacting stem cell differentiation [46]. The positively charged LDH nanoparticles employed in this study exhibited a strong affinity for the negatively charged cell membrane, facilitating their interaction with membrane receptors [28,29,47]. This interaction plays a crucial role in modulating the fate of stem cells. To elucidate the underlying mechanisms by which LDH nanoparticles promoted NPCs differentiation, it was imperative to verify the interactions between LDH nanoparticles and specific membrane receptors.

**Fig. 5:**
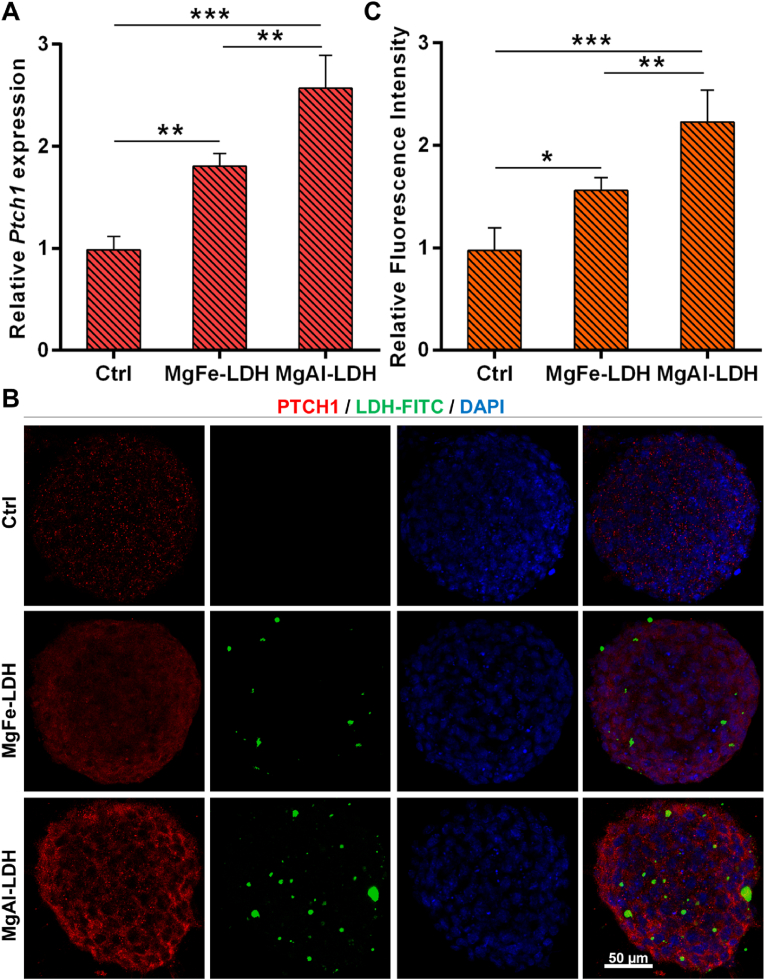
LDH enhanced NPCs differentiation by activating the Hedgehog signaling pathway through its interaction with the transmembrane protein PTCH1. A) qPCR detects the mRNA level of *Ptch1* in NPCs treated with nanoparticles; B) Colocalization of nanoparticles and PTCH1; C) The relative fluorescence intensity of PTCH1 in Fig. 5B. The data are presented as the mean ± SD (n = 3, ∗∗∗p < 0.001, ∗∗p < 0.01, ∗p < 0.05).

Based on the RNA-seq results, we discovered that LDH may activate the hedgehog signaling pathway by binding to the transmembrane protein PTCH1, thereby promoting the differentiation of NPCs. As shown in Fig. 5A, both MgFe-LDH and MgAl-LDH significantly upregulated the mRNA levels of Ptch1, with the MgAl-LDH group exhibiting higher Ptch1 mRNA levels compared to the MgFe-LDH group. Subsequently, we examined the colocalization of MgFe-LDH and MgAl-LDH with PTCH1 using confocal microscopy. Immunofluorescence results indicated that, both MgFe-LDH and MgAl-LDH showed colocalization with PTCH1, and MgAl-LDH group possessed stronger PTCH1 fluorescence intensity compared to the MgFe-LDH group (Fig. 5B and C).

The surface of LDH nanoparticles exhibits positive charges, while specific domains of PTCH1 demonstrates negative charge characteristics. This complementary charge distribution facilitates mutual approach and binding between LDH and PTCH1 through electrostatic attraction. The LDH surface contains abundant hydroxyl (-OH) groups and nitrate (NO_3_^−^) anions, which can form hydrogen bonds with functional groups (e.g., amino and carboxyl groups) on receptor surfaces. As a relatively strong intermolecular force, hydrogen bonding significantly enhances both binding stability and specificity in the LDH-receptor interaction system. Notably, molecular dynamics simulations by Wang et al. revealed a remarkably strong total electrostatic interaction energy of −11010.0 kJ mol^−1^ between PTCH1 and LDH nanoparticles [18], indicating exceptionally stable binding through electrostatic interaction. While Shh is the natural ligand for PTCH1, LDH nanoparticles are chemically stable, non-immunogenic, and avoid rapid degradation issues common with protein ligands like Shh. LDH binded PTCH1 through electrostatic interactions and hydrogen bonding [18], with stronger and more sustained contact, ensuring prolonged pathway activation.

**Fig. 6:**
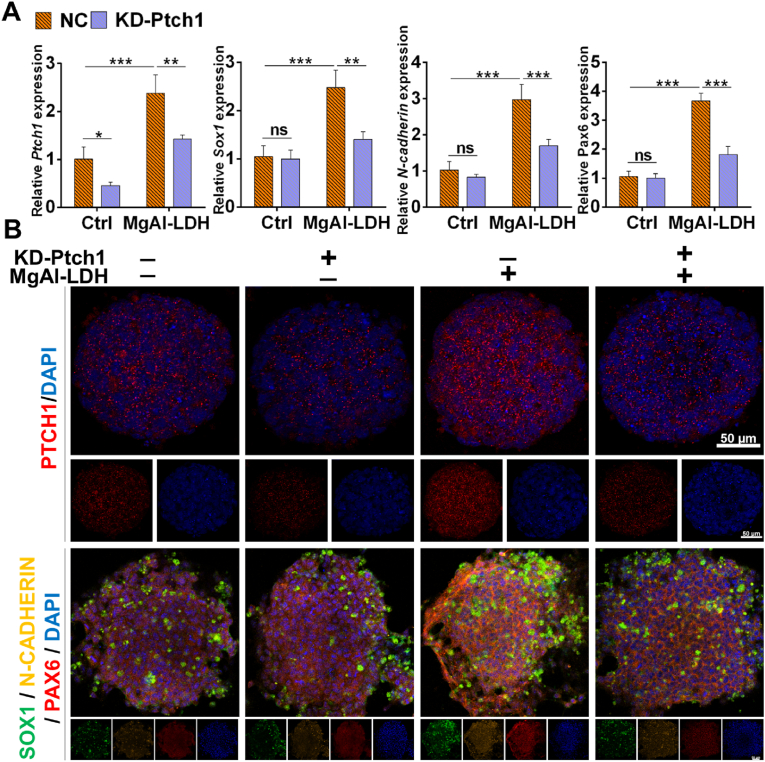
The effect of *Ptch1* deficiency on improved NPCs differentiation by MgAl-LDH. A) qPCR detects the effect of *Ptch1* deficiency on the mRNA level of *Sox1*, *N-cadherin*, and *Pax6* in NPCs treated with MgAl-LDH; B) Immunofluorescence detects the effect of *Ptch1* deficiency on the protein level of SOX1, N-CADHERIN, and PAX6 in NPCs treated with MgAl-LDH. The data are presented as the mean ± SD (n = 3, ∗∗∗p < 0.001, ∗∗p < 0.01, ∗p < 0.05, ns means there was no significant difference between the two groups).

The plasmid designed by Generay Biotechnology was employed to knockdown *Ptch1* expression in NPCs. qPCR analysis revealed that the mRNA level of *Ptch1* was significantly reduced in the KD-Ptch1 group compared to the NC group (Fig. 6A). Furthermore, the mRNA expression levels of NPCs markers, including *Sox1*, *Pax6*, and *N-cadherin*, were markedly diminished in the MgAl-LDH-KD-Ptch1 group relative to the MgAl-LDH-NC group (Fig. 6A). Compared to the MgAl-LDH-NC group, the reduced fluorescence intensity of these markers (SOX1, PAX6, and N-CADHERIN) in the MgAl-LDH-KD-Ptch1 group further corroborated that the promotive effect of MgAl-LDH on NPCs differentiation was attenuated by *Ptch1* deficiency, as illustrated in Fig. 6B and S4. In summary, the above results indicated that LDH promoted NPCs differentiation by activating hedgehog signaling pathways through interaction with the transmembrane protein PTCH1. Compared to MgFe-LDH, MgAl-LDH exhibited a higher affinity for binding with PTCH1, thereby more effectively activating hedgehog signaling pathways and facilitating NPCs differentiation.

### 3.6 LDH-pretreated NPCs transplantation promoted functional recovery in SCI mice

Stem cell transplantation has demonstrated promising outcomes in facilitating neural regeneration and improving neurological function, suggesting its significant potential in the clinical treatment of spinal cord injury [48]. Stem cells, endowed with the capacity for differentiation and the secretion of various growth factors, play a crucial role in providing nutritional support, promoting axonal regeneration, and neural repair [49]. However, the inflammatory microenvironment in the SCI region leads to low cell survival rates and challenges in controlling differentiation directions, with transplanted stem cells primarily differentiating into astrocytes or remaining in an undifferentiated state. Therefore, pre-differentiating transplanted cells into progenitor cells can enhance their utilization and control their differentiation direction [50]. NPCs derived from stem cells are capable of self-renewal and differentiation into all types of neural cells, and they can migrate and integrate into damaged areas, exhibiting high survival rates and plasticity in neural transplantation [51].Jessica et al. differentiated pluripotent stem cells into precursors of specific neurons (V2a interneurons) in vitro and found that the transplanted cells could effectively establish neural networks, promote the reconstruction of neuronal circuits, and integrate into damaged synaptic connections [52]. Ken et al. transplanted NPCs into an SCI rat model and observed the promotion of axonal growth and remyelination, improving the functionality of forelimb movement-related synapses in rats [53]. NPCs required for SCI transplantation are generally sourced from ESCs or induced pluripotent stem cells (iPSCs). iPSCs carry the epigenetic marks of the original cells after reprogramming, while ESCs possess genomic stability and lack exogenous genetic imprints. Inducing differentiation into precursors of specific neurons can effectively avoid drawbacks such as tumorigenicity and immune rejection [54,55].

**Fig. 7:**
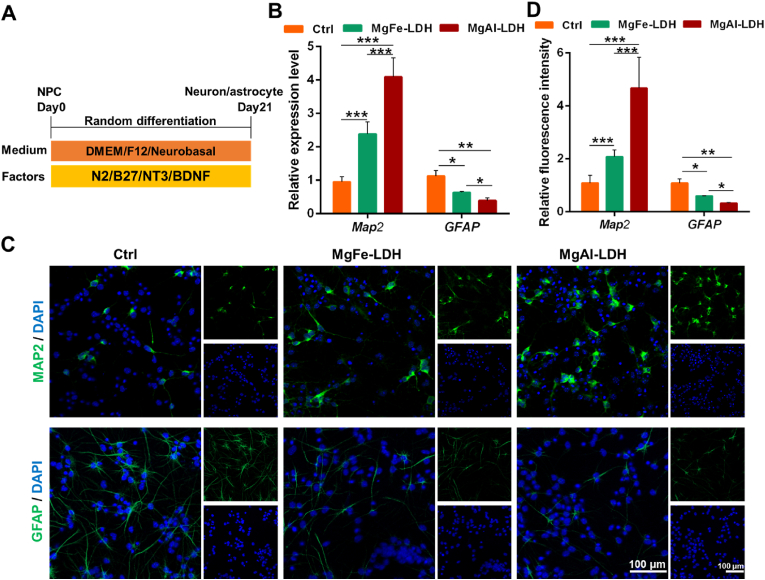
Spontaneous differentiation of LDH-treated NPCs. (A) Strategic diagram of the spontaneous differentiation of NPCs. (B) mRNA level of Map2 and GFAP in the indicated differentiated cells at day 21. (C) Immunofluorescence analysis of the protein level of MAP2 and GFAP in the indicated differentiated cells at day 21. (D)The relative fluorescence intensity of MAP2 and GFAP in Fig. 7C. The data are presented as the mean ± SD (n = 3, ∗∗∗p < 0.001, ∗∗p < 0.01, ∗p < 0.05).

Given the pivotal role of neuronal differentiation from NPCs in SCI physiological recovery, we further performed spontaneous differentiation of LDH-treated NPCs to evaluate the effectiveness of LDH in promoting neuronal differentiation (Fig. 7A). After 21 days of culture, the expression levels of neuronal marker (MAP2) exhibited significant upregulation in the LDH-treated groups compared to the control group. Conversely, astrocyte marker (GFAP) expression showed a concomitant reduction in LDH-treated NPCs, indicating suppressed astrocyte differentiation. Notably, comparative analysis between MgAl-LDH and MgFe-LDH demonstrated superior neuronal differentiation efficacy of MgAl-LDH, possessing higher MAP2 expression and lower GFAP levels relative to MgFe-LDH (Fig. 7B–D).

**Fig. 8:**
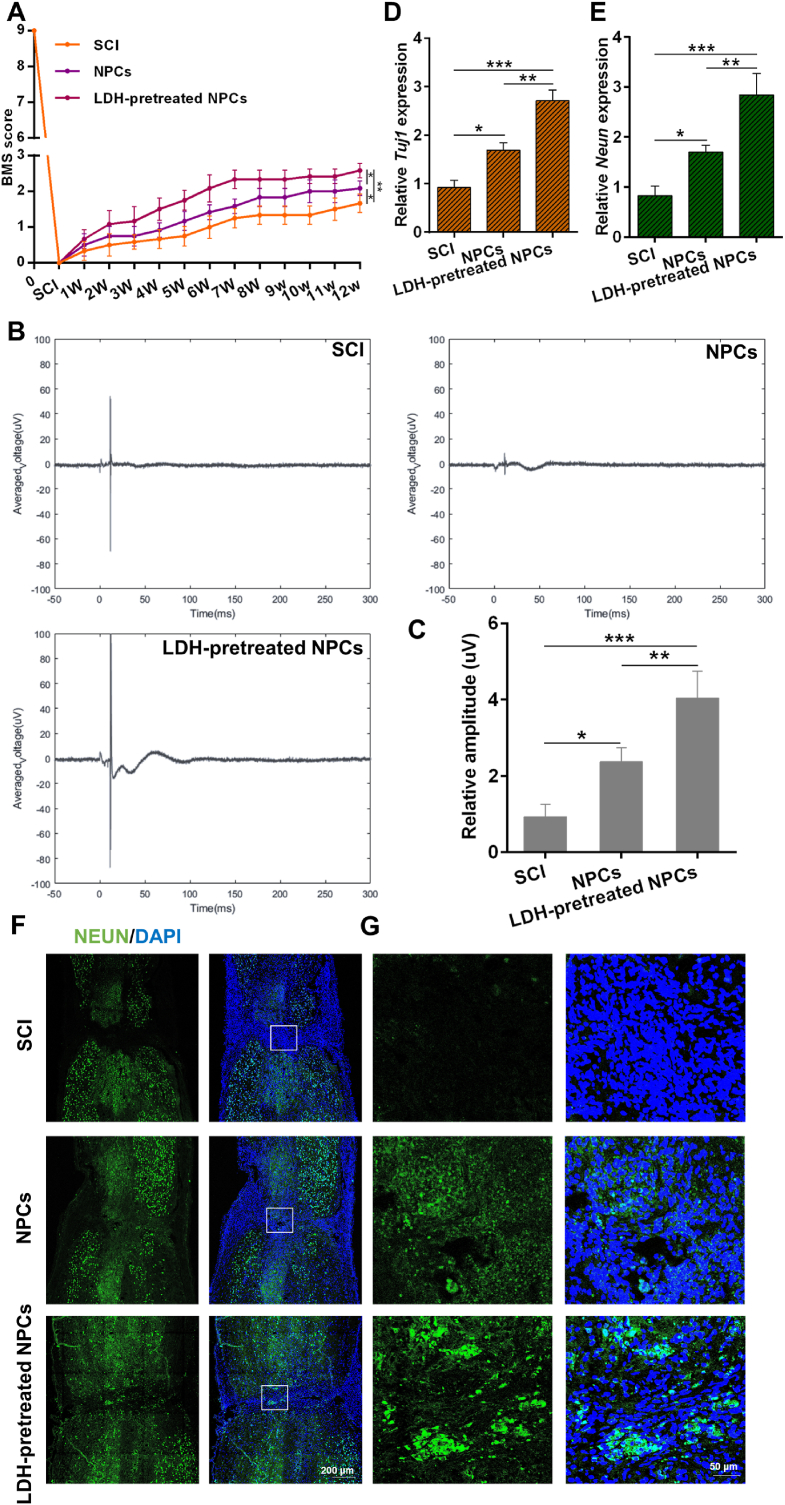
MgAl-LDH-pretreated NPCs promoted the recovery of motor function and neurogenesis in SCI mice. A) BMS open-field walking scores of bilateral hindlimbs for all the groups over time up to 12 weeks (n = 5, ∗∗p < 0.01, ∗p < 0.05); B) Electrophysiological analysis of SCI mice at the 12-week; C) Amplitude of MEPs; (n = 3, ∗∗∗p < 0.001, ∗∗p < 0.01, ∗p < 0.05); D,E) mRNA level of neural markers in the injured spinal cord in each group at the 12-week; (n = 3, ∗∗∗p < 0.001, ∗∗p < 0.01, ∗p < 0.05); F) Immunofluorescence staining of spinal cord sections 12-week after SCI; G) Amplified images of the lesion site in each group. The data are presented as the mean ± SD.

The above results indicated that MgAl-LDH was more efficient in promoting the neuronal differentiation of NPCs in vitro. To ascertain the therapeutic potential in vivo, mice with SCI were administered transplants of NPCs that had been pretreated with LDH. The locomotor functional recuperation of these SCI mice was quantitatively assessed using the Basso Mouse Scale (BMS), as depicted in Fig. 8A. At 12 weeks, the groups treated with NPCs and LDH-pretreated NPCs demonstrated a significant enhancement in functional recuperation compared to SCI group. Notably, the LDH-pretreated NPCs group exhibited the most pronounced recovery, with a BMS score of 2.25, signifying the superior functional recuperation among the evaluated groups. Subsequently, as illustrated in Fig. 8B and C, electrophysiological assessments were conducted to evaluate neural conduction by stimulating the cerebral cortex and capturing the hind limb motor evoked potential (MEP). Compared with the other two groups, the amplitude of LDH-pretreated NPCs group increased significantly, suggesting that LDH-pretreated NPCs transplantation more effectively facilitated neural conduction in SCI mice. Furthermore, we investigated whether the LDH-pretreated NPCs transplantation could stimulate neurogenesis in SCI mice. The expression levels of neuronal markers *Tuj1* and *Neun* were determined by qPCR. The results showed that the mRNA level of *Tuj1* and *Neun* were significantly elevated in NPCs and LDH-pretreated NPCs groups, and LDH-pretreated NPCs group possessed higher mRNA level of *Tuj1* and *Neun* than NPCs group (Fig. 8D and E). As presented in Fig. 8F and G and S5, NEUN signals were observed in both NPCs and LDH-pretreated NPCs groups, indicative of neurogenesis, and LDH-pretreated NPCs group possessed more NEUN signals than NPCs group. Fig. S6 depicted that LDH-pretreated NPCs group possessed a decreased expression of the M1 markers iNOS and TNFα and increased expression of the M2 marker IL10 and Arg-1 compared to SCI and NPCs groups, implying that the inflammatory effect was significantly inhibited. Fig. S7 demonstrated successful persistence of transplanted LDH-pretreated NPCs in the lesion region for up to 12 weeks post-transplantation. As depicted in Fig. S8, a comparative analysis of tissue morphology in the SCI, NPCs, and LDH-pretreated NPCs groups revealed no significant disparities, suggesting that the therapeutic interventions were non-toxic and potentially suitable for translation into clinical practice. Collectively, these results implied that LDH-pretreated NPCs transplantation could ameliorate motor functional recuperation and foster neurogenesis following SCI.

## 4 Conclusion

**Fig. 9:**
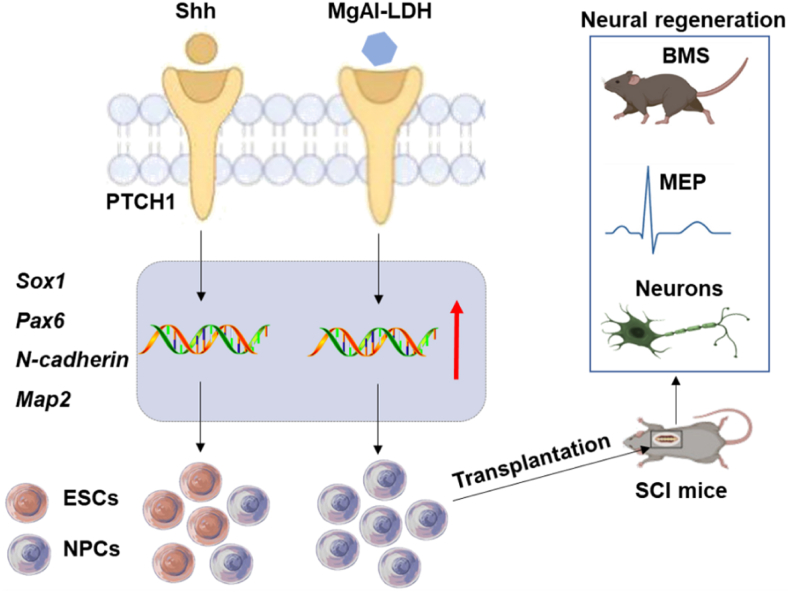
Schematic diagram of the repair of SCI by MgAl-LDH-pretreated NPCs.

In this study, we compared the effects of two different elemental LDH on the fate of NPCs, uncovering a novel function of LDH in stimulating NPCs differentiation. Our results demonstrated that MgAl-LDH significantly outperformed MgFe-LDH in promoting NPCs differentiation. RNA sequencing revealed that LDH activated hedgehog signaling pathways by regulating the expression of the transmembrane protein PTCH1. MgAl-LDH nanoparticles significantly upregulated the level of cell membrane receptor PTCH1 compared to MgFe-LDH, thereby significantly enhancing the NPCs differentiation. Furthermore, we found that knocking down PTCH1 significantly suppressed the promoted NPCs differentiation by MgAl-LDH. In *vivo*, MgAl-LDH-pretreated NPCs implantation significantly improved the behavioral and electrophysiological performance of SCI mice, and promoted neural regeneration in the lesion sites (Fig. 9). By comprehensively elucidating the role of LDH in modulating NPCs differentiation, we aimed to develop a highly efficient nanomaterial-mediated system for ESCs-based NPCs differentiation to repair spinal cord injury. This work deepens the insight into LDH's role in stem cell differentiation and presents an innovative approach for spinal cord injury recovery, divergent from previous applications of LDH in drug delivery and tissue engineering.

## CRediT authorship contribution statement

**Feng Zhang:** Writing – review & editing, Writing – original draft, Software, Methodology, Investigation, Funding acquisition. **Xinghao Pan:** Writing – review & editing, Supervision, Methodology, Investigation, Data curation. **Kaikai Zhang:** Writing – review & editing, Investigation, Data curation. **Shuhan Liu:** Visualization, Validation. **Danni Yu:** Supervision, Software. **Jingjing Su:** Software, Resources, Project administration. **Tong Zhu:** Supervision, Resources, Project administration, Funding acquisition, Formal analysis, Data curation. **Song Chen:** Writing – review & editing, Visualization, Validation, Software, Resources, Project administration, Methodology, Investigation, Funding acquisition, Formal analysis.

## Declaration of competing interest

The author declare that they have no competing interests.

## Data Availability

No data was used for the research described in the article.
