# Supplementary material for: Enhancing spinal cord injury repair through PTCH1-mediated neural progenitor cell differentiation induced by ion elemental-optimized layered double hydroxides
Source: Mater Today Bio. 2025 May 29;32:101918. doi: 10.1016/j.mtbio.2025.101918 (PMC12169790; doi:10.1016/j.mtbio.2025.101918)
Supplement: Multimedia component 1 [file mmc1.docx]

SUPPORTING INFORMATION

# Enhancing spinal cord injury repair through PTCH1-mediated neural progenitor cell differentiation induced by ion elemental-optimized layered double hydroxides

Feng Zhang^1, #^, Xinghao Pan^6, #^, Kaikai Zhang^1, #^, Shuhan Liu^5^, Danni Yu^5^, Jingjing Su^4, *^, Tong Zhu^3, *^, Song Chen^2, *^

^1^ Department of Orthopedics, Centre for Leading Medicine and Advanced Technologies of IHM, The First Affiliated Hospital of USTC, Division of Life Sciences and Medicine, University of Science and Technology of China, Hefei, Anhui, 230001, China

^2^ Department of Orthopedics, Quzhou People's Hospital, The Quzhou Affiliated Hospital of Wenzhou Medical University, No.100, Minjiang Avenue, Quzhou,324000, Zhejiang, China

^3^ Shanghai Pulmonary Hospital, School of Medicine, Tongji University, Shanghai 200433, China

^4^ The First Affiliated Hospital of Anhui Medical University, Hefei, 230001, China

^5^ People's Hospital of Qianxinan Prefecture, Guizhou province, 562400, China

^6^ Department of Medicine, Lady Davis Institute-Jewish General Hospital, McGill University, Montreal, QC, Canada

^#^ These authors contributed equally to this work.

* Authors to whom any correspondence should be addressed.

Corresponding authors:

Jingjing Su, [2478813407@qq.com](mailto:2478813407@qq.com); Tong Zhu, [TongZ120@163.com,](mailto:TongZ120@163.com,) Song Chen, chensong89@126.com





**Fig. S1.** The relative fluorescence intensity of NPCs markers in Figure 3F (n=3, ***p < 0.001, **p < 0.01, *p < 0.05).


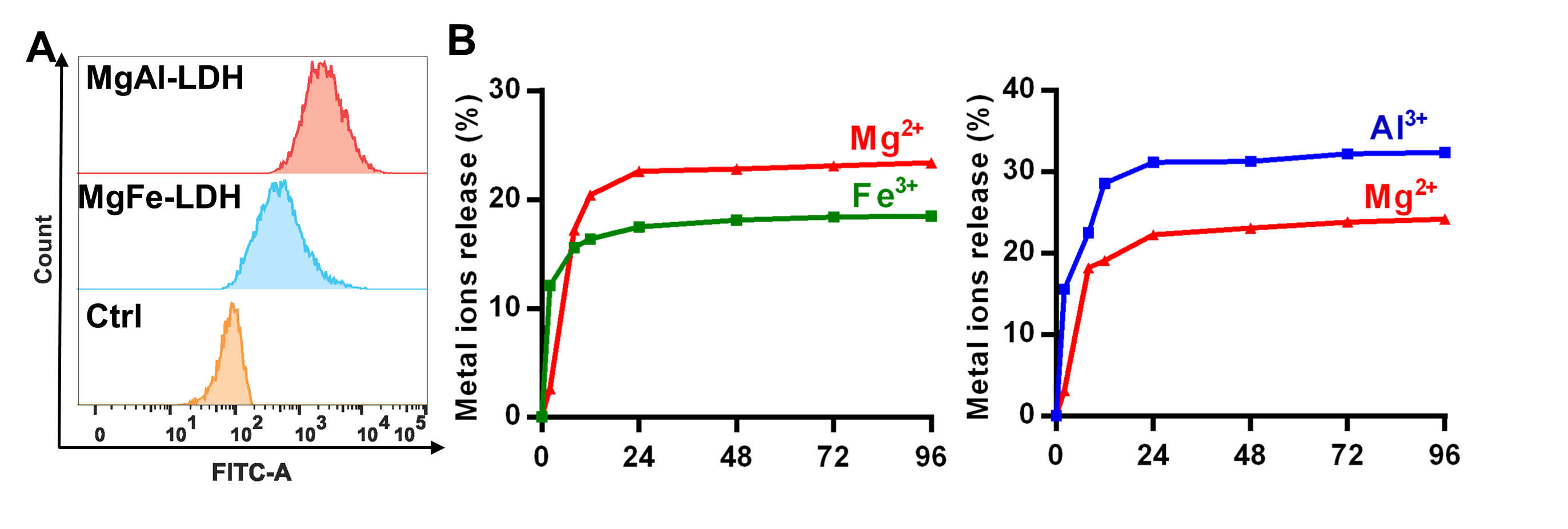


**Fig. S2.** (A) Flow cytometry analysis of cellular uptake of MgAl–LDH and MgFe–LDH in NPCs after 24 h incubation. (B) Metal ion release of MgAl–LDH and MgFe–LDH in PBS after different time.


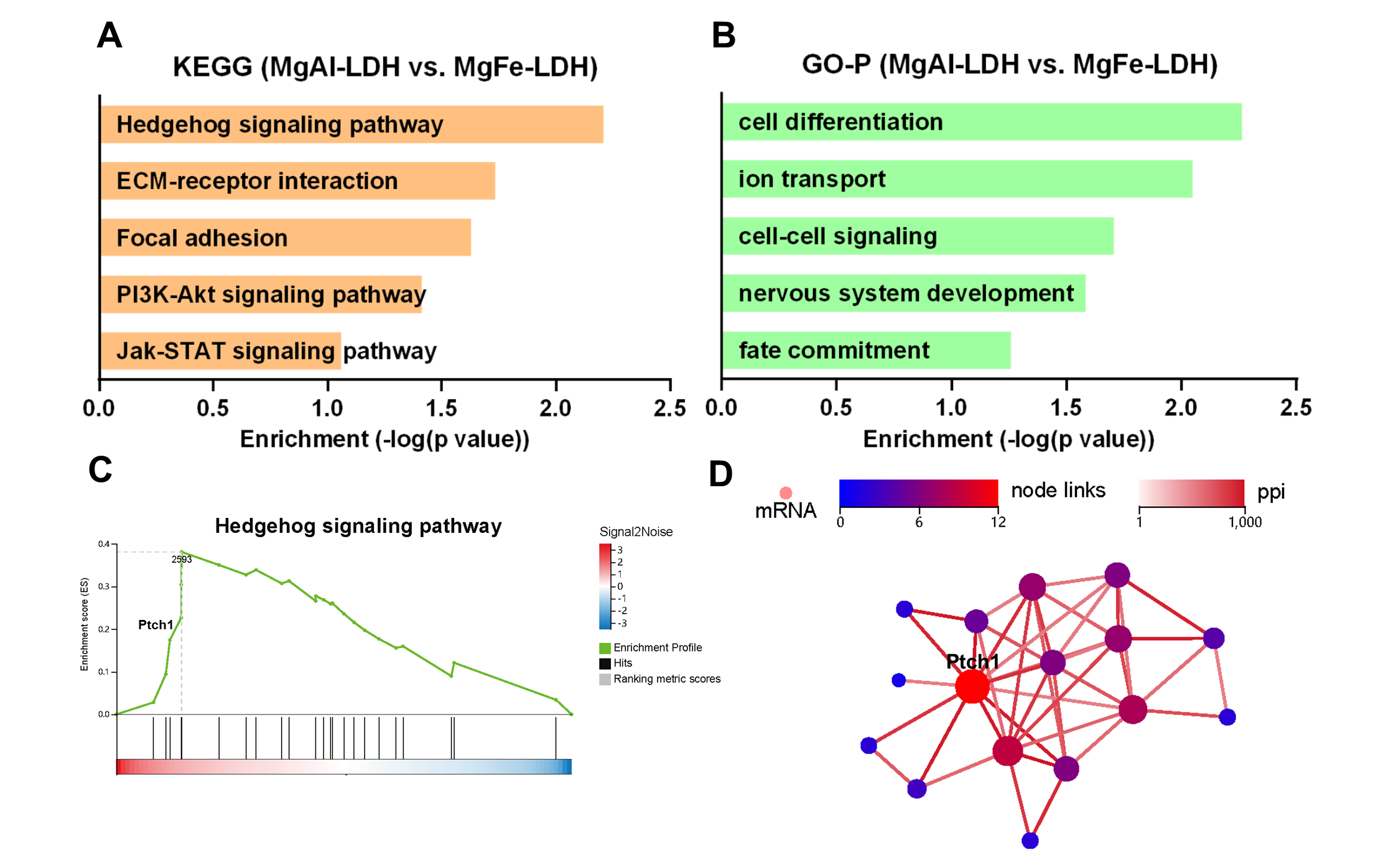


**Fig. S3.** Hedgehog signaling pathway differs significantly between the MgFe-LDH and MgAl-LDH groups, as identified via transcriptomic analysis. (A) KEGG pathway enrichment analyses. (B) GO enrichment analyses. (D) GSEA between the MgAl-LDH group and MgFe-LDH group (E) PPI analysis of MgAl-LDH regulated significant genes.





**Fig. S4.** The relative fluorescence intensity of PTCH1 and NPCs markers in Figure 6B (n=3, ***p < 0.001, **p < 0.01, *p < 0.05, ns means there was no significant difference between the two groups).





**Fig. S5.** Quantification of NEUN positive neural cells of lesion site in Figure 8G (n=3, ***p < 0.001, **p < 0.01).



**Fig. S6.** mRNA analysis of M1 and M2 markers in SCI mice.


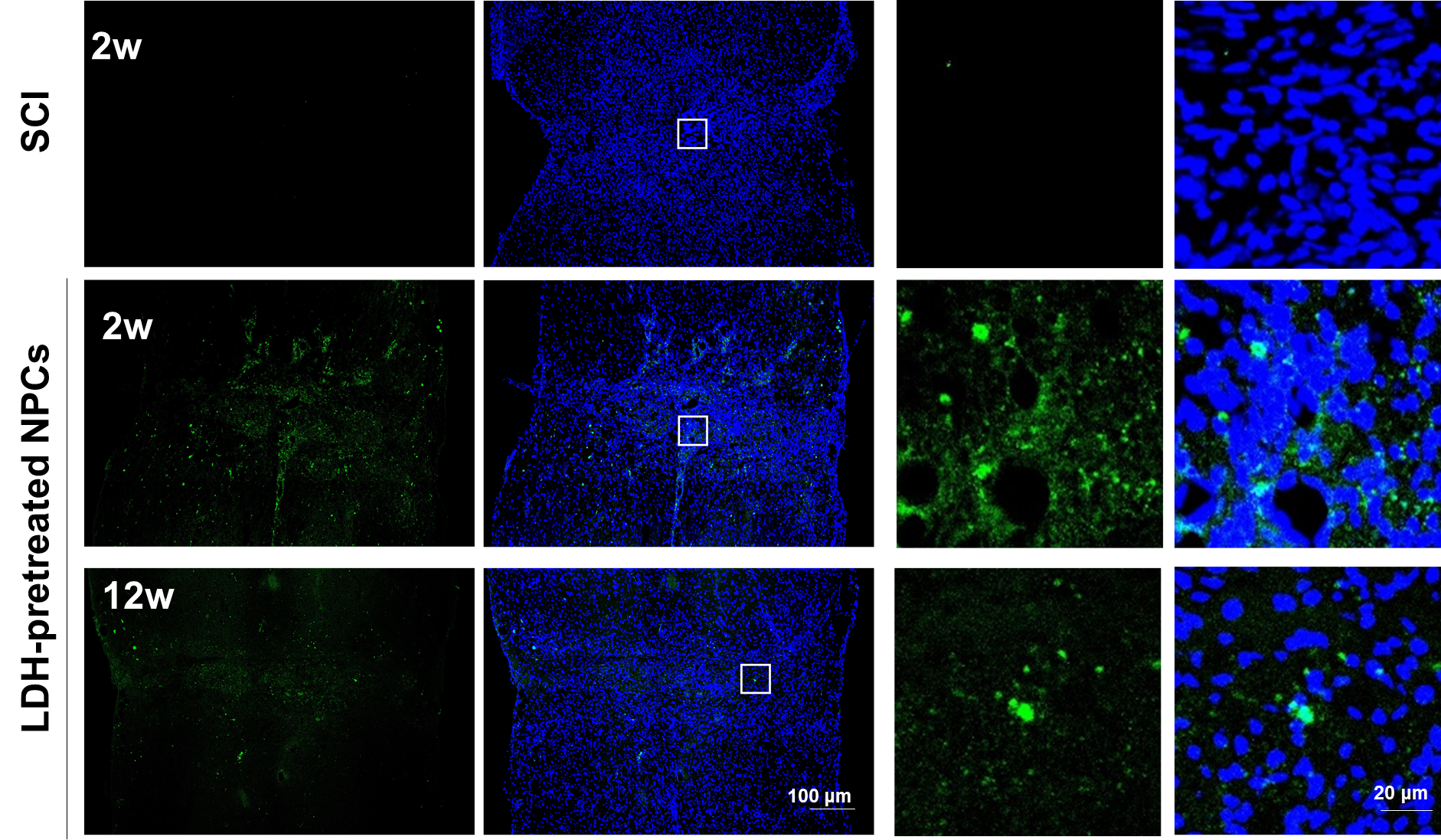


**Fig. S7.** The sustained survival of LDH-pretreated NPCs labeled by GFP in the lesion region.


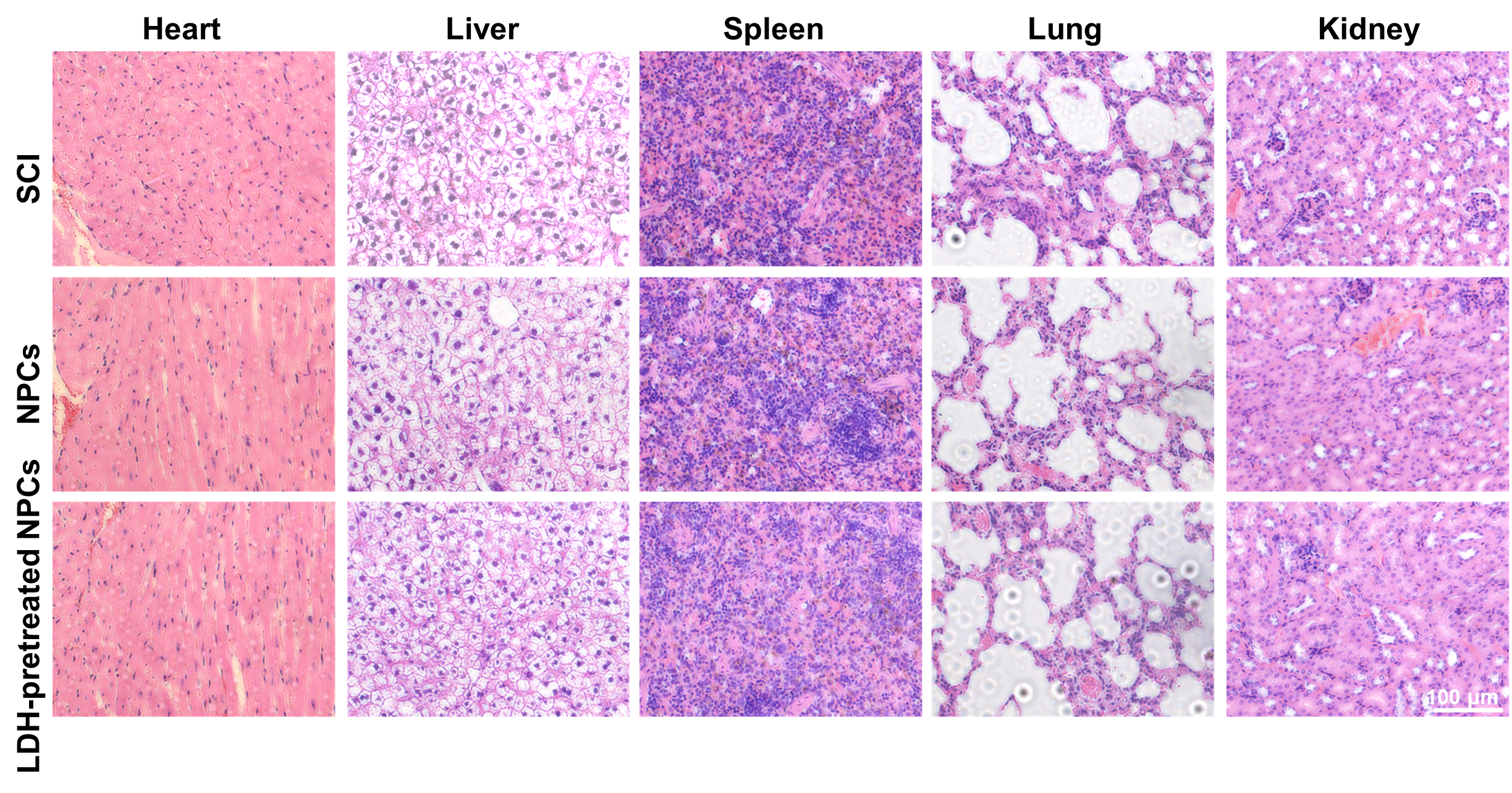


**Fig. S8.** H&E staining of the tissue in all groups.

| Gene name | Forward primer (5’-3’) | Reverse primer (5’-3’) |
| --- | --- | --- |
| *Gapdh* | GTGTTCCTACCCCCAATGTGT | ATTGTCATACCAGGAAATGAGCTT |
| *Sox1* | TTACTTCCCGCCAGCTCTTC | TGATGCATTTTGGGGGTATCTCTC |
| *Pax6* | TCTTTGCTTGGGAAATCCG | CTGCCCGTTCAACATCCTTAG |
| *N-cadherin* | TCCTGATATATGCCCAAGACAA | TGACCCAGTCTCTCTTCTGC |
| *Map2* | GGTCACAGGGCACCTATTCA | TGTTCACCTTTCAGGACTGC |
| *GFAP* | CCCTGGCTCGTGTGGATTT | GACCGATACCACTCCTCTGTC |
| *TNFα* | ATGCTGGGACAGTGACCTGG | CCTTGATGGTGGTGCATGAG |
| *iNOS* | ATCTTTGCCACCAAGATGGCCTGG | TTCCTGTGCTGTGCTACAGTTCCG |
| *IL10* | GCTGGACAACATACTGCTAACC | ATTTCCGATAAGGCTTGGCAA |
| *Arg-1* | CTCCAAGCCAAAGTCCTTAGAG | AGGAGCTGTCATTAGGGACATC |
| *Ptch1* | TGTTCCAGTTAATGACTCCC | ACACTCTGATGAACCACCTC |

Table S1. qPCR primer.
